# Childhood triglyceride-glucose index and pre-hypertension in adulthood: a prospective cohort study

**DOI:** 10.3389/fendo.2025.1489325

**Published:** 2025-04-14

**Authors:** Lingli Zhao, Zhijie Cui, Jiahui Ouyang, Hua Qu, Zhuye Gao

**Affiliations:** ^1^ Xiyuan Hospital, China Academy of Chinese Medical Sciences, Beijing, China; ^2^ National Clinical Research Center for Chinese Medicine Cardiology, Beijing, China

**Keywords:** triglyceride-glucose index, pre-hypertension, race, gender, cohort study

## Abstract

**Background:**

The triglyceride-glucose (TyG) index serves as a surrogate marker for insulin resistance. Multiple studies have demonstrated a positive correlation between the TyG index and blood pressure, indicating that a high TyG index is related to a greater risk of developing pre-hypertension (pre-HTN) and hypertension (HTN). However, the relationship between changes in the TyG index during childhood and pre-HTN in adulthood requires further clarification.

**Methods:**

The present prospective study utilized data from the Bogalusa Heart Study, a long-term follow-up study. Data on triglycerides (TG), fasting glucose (Fg), and low-density lipoprotein cholesterol (LDL-C) were collected from cross-sectional examinations of participants during childhood. Blood pressure (BP) in early adulthood was categorized into normotensive and pre-HTN groups. Logistic regression was employed to evaluate the relationship between the TyG index in childhood and pre-HTN in adulthood.

**Results:**

A total of 1,222 participants were included in the study, of whom 258 presented with pre-HTN in adulthood. Significant differences were observed in baseline TyG index, body mass index (BMI), and high-density lipoprotein cholesterol (HDL-C) between the two groups. In both unadjusted logistic regression (Odds Ratio (OR):1.8, 95% CI: 1.4, 2.5, *P* < 0.001) and simple adjustment (OR: 1.7, 95% CI: 1.2, 2.3, *P* = 0.003), childhood TyG index were significantly associated with pre-HTN in adulthood. However, this significant relationship disappeared after full adjustment (OR: 1.2, 95% CI: 0.8, 1.9, *P* = 0.373) which extended Model 1 by including adjustments for baseline BMI, baseline HDL-C, baseline LDL-C, smoking status, drinking status, use of antihypertensive medication and family history of HTN.Stratified analysis in Model 2 showed that gender and race significantly affected the relationship between TyG index and BP. In the male population, elevated TyG index levels increased the probability of pre-HTN, whereas no such relationship was found in female (Male: OR: 1.9, 95% CI: 1.1, 3.5, P = 0.029; Female: OR: 0.8, 95% CI: 0.4, 1.4, P = 0.447; P for interaction = 0.037). Similarly, in American Caucasians, TyG was positively associated with the risk of pre-HTN, but this relationship was not observed in African American (American Caucasian: OR: 1.7, 95% CI: 1.0, 2.9, *P* = 0.035; African American: OR: 0.5, 95% CI: 0.2, 1.1, *P* = 0.087; *P* for interaction = 0.007).

**Conclusions:**

In males and Caucasians, elevated TyG index during childhood can increase the risk of pre-HTN in adulthood. Monitoring the TyG index may help in screening individuals at higher risk of pre-HTN.

## 1 Introduction

Cardiovascular events result in over 18 million deaths annually, constituting approximately one-third of global deaths (1). HTN impacts nearly a quarter of the global population and stands as a significant modifiable risk factor for cardiovascular disease (CVD) and mortality (2). Studies have shown that pre-HTN has a high incidence in both developed and developing countries. In China, 41.3% of the adult population aged 18 years or older (approximately 435.3 million people) has pre-HTN (3); Among respondents aged 18-49 years, the prevalence of pre-HTN in India, Bangladesh, and Nepal was 43.2%, 35.1%, and 25.2%, respectively (4); A meta-analysis of 1,312,244 participants from the Middle East and North Africa found a pre-HTN prevalence of 30.6% (5); Additionally, 28.2% of the 30,958 U.S. adults aged 20 years or older who participated in the National Health and Nutrition Examination Survey in 2011-2012 had pre-HTN (6); In the Southern Cone of Latin America, it is estimated that 32.5% of the study population had pre-HTN (7).

Patients with pre-HTN are associated with a risk of cardiovascular disease, and much of this risk is attributable to the fact that people with pre-HTN have a high probability of developing HTN (8). The prevalence of HTN among young adults continues to rise (9), but commonly used risk prediction models or guidelines are predominantly based on studies of middle-aged and older adults (10–12). Therefore, further studies are needed to supplement the risk prediction of HTN in young adulthood to support clinical diagnosis and to help evaluate the safety and effectiveness of antihypertensive therapy in this population. The TyG index, a composite measure of fasting TG and Fg, is calculated as TyG index = ln [TG (mg/dl) × Fg(mg/dl)/2] (13). It has been recognized as a reliable biomarker of insulin resistance (IR) (14). Multiple studies have provided statistical evidence supporting a strong association between the TyG index and the development and prognosis of pre-HTN (15–17). Insulin resistance can lead to increased blood pressure through a number of pathways. It promotes the absorption of glucose and sodium by the proximal convoluted tubules (18, 19), stimulates sympathetic nerve activity, increases renin secretion, and attenuates nitric oxide (NO)-mediated vasodilation (20, 21), Additionally, it promotes vascular remodeling and arterial stiffness (22–24), which increases blood volume and vascular resistance, eventually leading to elevated BP.

Consequently, pre-HTN serves as an important early warning signal for future HTN and the associated risks of cardiovascular and cerebrovascular diseases, including death. Early intervention is crucial to reduce these risks and delay or prevent disease progression. The TyG index can be utilized as an early warning tool to identify high-risk individuals, allowing for effective lifestyle adjustments, regular monitoring, and individualized health management to slow or prevent the onset of HTN or other major diseases. This study, based on data from the Bogalusa Heart Study—a long-term follow-up project available in the BioLINCC database—explores the relationship between the TyG index and BP in early adulthood. It highlights the value of the TyG index trajectory in childhood for predicting pre-HTN in adulthood and examines its potential limitations as a predictor, providing broader and more accurate supporting evidence.

## 2 Materials and methods

### 2.1 Population and study design

This study cohort is derived from the Bogalusa Heart Study, which is a longitudinal study initiated in 1973 in Bogalusa, Louisiana, USA. This relatively small town has a significant African American and American Caucasian population. The study has conducted seven childhood cross-sectional exams and one adulthood exam, the latter referred to as Z510. Individuals who participated in the Z510 adulthood exam had also participated in at least one childhood exam. For this analysis, we used data from the Z510 exam and the participants’ first childhood exam. In order to minimize the impact of childhood blood pressure levels on adult blood pressure, we excluded individuals who had hypertension during childhood or adolescence, in accordance with the definition of hypertension in children and adolescents outlined in the *Clinical Practice Guideline for Screening and Management of High Blood Pressure in Children and Adolescents* (25).

**Figure 1:**
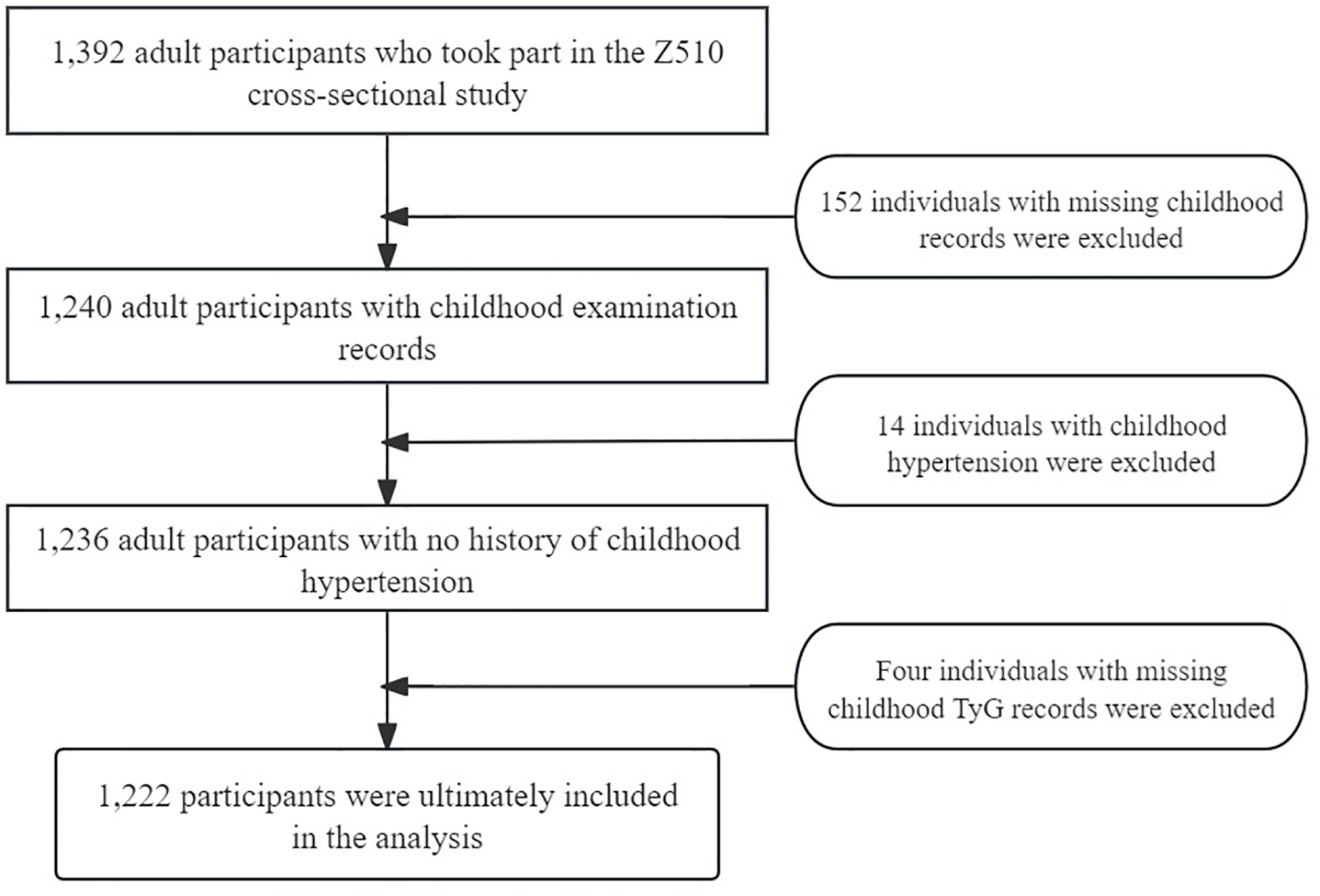
Flowchart of the study sample.

In our study, we included both baseline and post-adulthood medication information. The baseline data covered whether the individuals were using medications for heart disease, hormonal contraceptives, insulin injections, or anticonvulsants. For the post-adulthood period, we specifically included whether the participants were taking antihypertensive medications. After excluding individuals who did not meet the inclusion criteria, the final analysis included 1,222 individuals. Among them, none used heart disease medications, hormonal contraceptives, or insulin during adolescence, and only six individuals used anticonvulsants. The use of anticonvulsants did not show a statistically significant association with elevated blood pressure in adulthood, so we did not include this factor in the final analysis. Instead, we focused on the current use of antihypertensive medications. Based on BP readings, these individuals were divided into two groups: normal BP and pre-HTN (**Figure 1**).

The study was authorized by the Tulane University Health Science Center’s Institutional Review Board after each participant or their legal guardian provided informed permission. The data analysis protocol was authorized by the Medical Ethics Committee of Xiyuan Hospital, China Academy of Traditional Chinese Medicine, and the necessary data was given by the National Heart, Lung, and Blood Institute (NHLBI).

### 2.2 Data collection and variables

The investigators were trained to collect data following established procedures. The subjects were required to abstain from eating for at least 8 hours before the examination. Questionnaires on smoking, alcohol use, medication usage, and other significant illnesses as well as information on cardiovascular and other conditions, were filled out in a calm, private setting. Those who had given up smoking for more than a month or who smoked fewer than one cigarette per week were classified as nonsmokers. Those who had not consumed alcohol for more than a year were considered nondrinkers. BP was measured using a mercury sphygmomanometer, with six readings taken from the right arm while the participant was seated. A registered nurse collected the blood samples, which were drawn from either the left or right arm, according to the patient’s preference and the availability of the arm. Physical measurements were recorded by experienced technicians using professional equipment.

Serum lipoprotein cholesterol and TG were measured using heparin calcium precipitation and agar gel electrophoresis with a Hitachi 902 automated analyzer (Roche Diagnostics, Indianapolis, Indiana). Fg were assessed enzymatically with a multichannel Olympus AU-5000 analyzer (Olympus, Lake Success, NY). Pre-HTN was defined according to the 2017 hypertension guidelines from the American Heart Association (AHA) and the American College of Cardiology (ACC) as having a systolic blood pressure (SBP) of 120-129 mmHg and/or a diastolic blood pressure (DBP) of 80-84 mmHg (26).

### 2.3 Statistical analysis

R v.4.2.0 (R Foundation; https://www.r-project.org/) and empowering stats software(R) (www.empowerststs.com; X & Y Solutions. Neneneba Inc.) were used to analyze BP. The χ² test and Kruskal-Wallis rank sum test were used to compare the characteristics between normotensive and hypertensive individuals.

Logistic regression assessed the relationship between the TyG index in childhood and pre-HTN in adulthood. Model 1 controlled for baseline age, gender, and race. Model 2 extended Model 1 by adjusting for baseline BMI, HDL-C, LDL-C, smoking status, drinking status, use of antihypertensive medication and family history of HTN. The threshold for statistical significance was set at a value of α < 0.05.

Stratified analyses assessed the heterogeneity of the association between TyG and pre-HTN among subgroups defined by gender, race, lipid levels, BMI, smoking status, drinking status, family history of HTN and use of antihypertensive medication. HDL-C was categorized into < 40 mg/dl, 40 - 60 mg/dl, and > 60 mg/dl groups; LDL-C into < 110 mg/dl, 110 - 130 mg/dl, and > 130 mg/dl groups; and BMI into < 5%, 5%-95%, and > 95% groups. Interaction terms were added to investigate the moderating effects of these factors.

## 3 Results

The study cohort consisted of a total of 1,222 participants, comprising 740 women (60.56%) and 354 African American individuals (28.97%), with a mean (SD) baseline age of 11.30 (4.00) years. Among these participants, 258 exhibited pre-HTN in adulthood, while 964 maintained normal BP, with a mean (SD) endpoint age of 29.0 (5.00) years. The characteristics of the participants are detailed in **Table 1**.

**Table 1:** Characteristics of study chorts by Pre-HTN.

Variables^a^	Without Pre-HTN N=964	With Pre-HTN N=258	*P* Value*
TyG	7.90 ± 0.40	8.00 ± 0.50	<0.001
Baseline age, y	10.98 ± 4.00	12.52 ± 3.91	<0.001
Endpoint age, y	28.50 ± 5.08	30.75 ± 4.60	<0.001
Endpoint SBP, mmHg	107.5 ± 7.9	124.4 ± 11.0	<0.001
Endpoint DBP, mmHg	70.3 ± 5.9	86.3 ± 6.5	<0.001
Use of antihypertensive medication, n(%)			<0.001
No, (%)	1112 (95.86%)	48 (4.14%)	
Yes, (%)	25 (62.50%)	15 (37.50%)	
Race			0.028
American Caucasian, n (%)	699 (80.53%)	169 (19.47%)	
African American, n (%)	265 (74.86%)	89 (25.14%)	
Gender			<0.001
Male, n (%)	331 (68.67%)	151 (31.33%)	
Female, n (%)	633 (85.54%)	107 (14.46%)	
HDL-C, mg/dL	60.75 ± 16.76	57.08 ± 20.38	0.003
LDL-C, mg/dL	87.03 ± 24.87	88.07 ± 24.51	0.551
BMI, kg/m^2^	18.13 ± 3.49	20.60 ± 5.31	<0.001
Smoking status^b^			0.890
No, (%)	484 (79.47%)	125 (20.53%)	
Yes, (%)	425 (79.14%)	112 (20.86%)	
Drinking status^b^			0.194
No, (%)	238 (76.77%)	72 (23.23%)	
Yes, (%)	699 (80.25%)	172 (19.75%)	
Family history of HTN^b^			0.003
No, (%)	462 (82.80%)	96 (17.20%)	
Yes, (%)	496 (75.73%)	159 (24.27%)	

* Kruskal-Wallis test was used for continuous variables and Fisher’s test for categorical variables. The chi-square test was used to compare the characteristics of the two groups.

a Except for endpoint age, all other indicators in the variables are derived from baseline data.

b The sample size used to calculate the statistics was smaller than the total sample size due to the lack of information on smoking status, alcohol consumption and family history of HTN for some adult participants.

Those with pre-HTN in adulthood had significantly higher baseline TyG index, baseline age and BMI levels compared to the normotensive group (TyG: 8.00 vs. 7.90; BMI: 20.60 vs. 18.13; Baseline age:12.52 vs. 10.98; *P* < 0.001). Their HDL-C levels were significantly lower (57.08 vs. 60.75; *P* = 0.003). Additionally, significant discrepancies were observed in the demographic characteristics of the two groups, including gender, race, Use of antihypertensive medication and family history of HTN.A higher proportion of Male than Female populations received pre-HTN (151 vs. 107; P < 0.001).American Caucasian had a higher rate of Pre-HTN acquisition than the African American population (169 vs. 89; P=0.028). Participants with antihypertensive medication had a lower rate of acquiring Pre-HTN in adulthood than those without antihypertensive medication (15 vs. 48; P < 0.001). Those with Family history of HTN had a higher rate of pre-HTN in adulthood (159 vs. 96; P = 0.003).

As presented in **Table 2**, unadjusted logistic regression (OR: 1.8, 95% CI: 1.4, 2.5, *P* < 0.001) and Model 1 (OR: 1.7, 95% CI: 1.2, 2.3, *P* = 0.003) revealed a significant relationship between the TyG index in childhood and pre-HTN in adulthood. However, after complete adjustment, this relationship became insignificant. (OR: 1.2, 95% CI: 0.8, 1.9, *P* = 0.373). Consequently, an interaction test was conducted to identify specific subpopulations.

**Table 2:** Adjusted means (95% CI) of childhood TyG levels and adult blood pressure.

Models	OR (95% CI)	*P* Value
Not adjusted	1.8 (1.4, 2.5)	<0.001
Model 1^*^	1.7 (1.2, 2.3)	0.003
Model 2^^^	1.2 (0.8, 1.9)	0.373

* Model 1 used logistic regression for participants with elevated and normal BP, controlling for baseline age, gender, and race.

^ Model 2 extended Model 1 by including adjustments for baseline BMI, baseline HDL-C, baseline LDL-C, smoking status, drinking status, use of antihypertensive medication and family history of HTN.

The interaction test revealed that gender, race, and smoking status had a significant impact on how TyG affected BP (**Table 3**). In males, a higher TyG index was linked to an increased risk of pre-HTN, a trend not seen in females. (Male: OR: 1.9, 95% CI: 1.1, 3.5, *P* = 0.029; Female: OR: 0.8, 95% CI: 0.4, 1.4, *P* = 0.447; *P* for interaction = 0.037). Among American Caucasians, a positive correlation was observed between TyG and the risk of pre-HTN which was absent in African American participants (American Caucasian: OR: 1.7, 95% CI: 1.0, 2.9, *P* = 0.035; African American: OR: 0.5, 95% CI: 0.2, 1.1, *P* = 0.087; *P* for interaction = 0.007). Elevated TyG index were also linked to increased BP in nonsmokers, a relationship not seen in smokers (Smokers: OR: 2.1, 95% CI: 1.1, 3.9, *P* = 0.022; Nonsmokers: OR:0.8, 95% CI: 0.4, 1.5, *P* = 0.485; *P* for interaction = 0.036).

**Table 3:** Association between TyG and elevated BP according to baseline characteristics.

Subgroups^*^	Pre-HTN OR (95% CI)	P Value	*P* Value for Interaction
Gender			0.037
Male	1.9 (1.1, 3.5)	0.029	
Female	0.8 (0.4, 1.4)	0.447	
Race			0.007
American Caucasian	1.7 (1.0, 2.9)	0.035	
African American	0.5 (0.2, 1.1)	0.087	
HDL-C, mg/dl			0.692
<40	1.6 (0.6, 4.3)	0.352	
40-60	1.0 (0.5, 2.0)	0.958	
>60	1.0 (0.5, 2.0)	0.990	
LDL-C, mg/dl			0.094
<110	1.3 (0.8, 2.1)	0.227	
110-130	0.3 (0.1, 1.2)	0.093	
>130	2.7 (0.4, 16.9)	0.286	
Smoke status			0.036
No	2.1 (1.1, 3.9)	0.022	
Yes	0.8 (0.4, 1.5)	0.485	
Drinking status			0.848
No	1.3 (0.5, 3.0)	0.564	
Yes	1.4 (0.8, 2.4)	0.183	
Family history of HTN			0.982
No	1.2 (0.6, 2.2)	0.613	
Yes	1.2 (0.7, 2.1)	0.602	
Use of antihypertensive medication			0.105
No	1.2 (0.8, 1.8)	0.422	
Yes	38.2 (0.3, 4346.3)	0.132	

*All factors (gender, race, HDL-C, LDL-C, BMI, smoke status, drinking status, use of antihypertensive medication, family history of HTN) were adjusted for in each stratification in addition to stratification factors.

## 4 Discussion

In this prospective cohort study based on the Bogalusa Heart Study, we identified a statistically significant association between an increase in TyG index in childhood and pre-HTN in adulthood, stratified by race and gender. Each unit increase in the TyG index was associated with a 70% increased likelihood of pre-HTN among American Caucasian participants and a 90% increased likelihood among male participants, independent of potential confounders. This suggests that a high TyG index may be a robust predictor of pre-HTN events in these groups. And we also found the increase in the TyG index was associated with a 110% increased likelihood of pre-HTN among non-smokers. A cross-sectional study of 32,124 adults with normal blood glucose found that an elevated TyG index was associated with pre-HTN and has the potential to be a cost-effective monitoring tool in the management of pre-HTN and HTN classification (15). A retrospective study involving 15,450 subjects found that the prevalence of pre-HTN increased with higher TyG index quintiles, highlighting the strong correlation between TyG index and pre-HTN in Japanese subjects with normal blood glucose (16). A cross-sectional survey of 3,115 subjects found that in Chinese adults, a higher TyG index independently and synergistically increases the risk of pre-HTN and is more predictable than traditional indices (17).

People with pre-HTN are a key target population for promoting lifestyle changes to prevent the progression to HTN and reduce the risk of cardiovascular disease. If this high-incidence pre-HTN population is not adequately managed, it may lead to a higher prevalence of clinical HTN, which, in turn, increases cardiovascular mortality (27). Although age, male sex, obesity, hypercholesterolemia, and diabetes mellitus are well-established risk factors for abnormal blood pressure, some individuals may develop HTN even in the absence of these factors (28). Therefore, identifying early risk groups for HTN is crucial for improving risk stratification and therapeutic management. By monitoring the trajectory of the TyG index from childhood, the likelihood of developing pre-HTN in adulthood can be predicted, allowing for early intervention to control and stabilize BP before it progresses to pre-HTN, thereby preventing further development into HTN and reducing associated cardiovascular risks. This proactive approach can significantly lessen the public health burden.

Stratifying by gender, our findings align with previous research, showing that elevated TyG index was more relevant for predicting pre- HTN in men compared to women (29). Visceral fat has a greater impact on impairment of insulin clearance than subcutaneous fat (30).The disparity in outcomes between male and female could be attributed to differences in adipose tissue distribution. Although women generally have a higher body fat content, men tend to accumulate more visceral fat (31), a phenomenon influenced by estrogen, which promotes adipose tissue deposition (32), enhances sympathetic action on adipose regions (33), and regulates vascular supply access to adipose tissue (34). Estrogen also affects the extensibility of adipose regions, favoring lipid accumulation in subcutaneous tissues in women and visceral fat deposition in men (35). Furthermore, differences in insulin sensitivity between males and females may be linked to mitochondrial dysfunction (36, 37), with evidence indicating potential variances in mitochondrial biology between African American and Caucasian males and females (38). Females exhibit higher levels of brown adipose tissue and increased expression of genes related to mitochondrial function, leading to higher metabolic rates per kilogram of adipose tissue than males (39, 40). Smoking is another crucial factor contributing to the heterogeneity between sexes, and the origin of the study population may partially explain the observed differences.

Stratifying by race, our results indicate that the TyG index is more predictive of pre-HTN risk in Caucasians, while it is not statistically significant in African Americans. This finding differs from the results of previous studies. Data from the Jackson Heart Study support the notion that higher IR is associated with an increased risk of higher insulin resistance levels are associated with greater risks of BP progression and incident hypertension among Community-based African American cohorts (41). The prevalence of HTN is higher in African American adults compared to non-Hispanic white or Hispanic adults (42). Furthermore, studies have found a higher incidence of e insulin resistant syndrome in African Americans compared to Caucasians (43). Therefore, assays that may distinguish between different insulin resistances are more relevant for predicting the incidence of pre-HTN in different races. Racial disparities in insulin sensitivity might be partly explained by differences in skeletal muscle mitochondrial function, muscle fiber composition, or the downstream effects of mitochondrial dysfunction (44, 45). Racial differences in the outcomes should not be viewed solely as biological constructs but rather in the context of socioeconomic, environmental, and systemic factors such as structural racism (46).Research suggests that childhood adversity and concurrent adulthood stresses, including socioeconomic adversity and discrimination, may influence IR through inflammatory and hypothalamic-pituitary-adrenal (HPA) axis pathways, contributing to differences between African Americans and European American (47). It has also been shown that non-Hispanic blacks with normal BP, as well as non-Hispanic whites and non-Hispanic blacks with pre-HTN, have reduced cutaneous sensory nerve-mediated vasodilatation as well as cutaneous endothelial NO-dependent vasodilatation compared with non-Hispanic whites with normal BP (48).

Stratifying by smoking or not, we found that an increase in TyG index was associated with an increase in BP in non-smokers, but there was no similar relationship in smokers. Studies have shown that smoking is a risk factor for both HTN (49)and IR (50). For adolescents to middle-aged individuals, smoking accelerates the BP amplification effect during the initial stage of HTN (51). Differences in nicotine metabolism between races and differences in nicotine dependence due to racial discrimination lead to differences in the effects of smoking as a risk factor on BP and IR (52–54). Therefore, it may be that the influence of smoking as a risk factor on peripheral vascular resistance and on body metabolism leads to no statistically significant effect of TyG index on BP increase in smoking population. Further large sample studies are needed to verify the generality and accuracy of these results.

An in-depth exploration of the significance of gender and ethnicity in predicting pre-HTN using the TyG index could enhance our understanding of HTN risk across different populations and improve personalized care. This knowledge could guide the development of more targeted preventive strategies and treatment regimens. For example, interventions might focus more on visceral fat management in men and metabolic rate modification in women. Future studies could also delve into the roles of estrogen and mitochondrial function in these processes. Regarding race, research could further explore the interplay between racial differences in IR, socioeconomic factors, and structural racism, examining in greater detail how these factors influence cardiovascular disease outcomes across various populations. In clinical practice, individualized interventions that account for racial and socioeconomic variables could enhance the effectiveness of HTN prevention and management strategies. Ultimately, this approach could address health disparities more effectively and optimize clinical outcomes.

The TyG index is an indicator of IR, which is characterized by reduced responsiveness of insulin-targeted tissues to physiological levels of insulin, necessitating higher-than-normal levels of insulin to maintain normal insulin function (55). BP is controlled by the relationship between circulating fluid volume and peripheral vascular resistance (56). Circulating fluid volume is regulated by blood volume and cardiac contractility. Blood volume is influenced by sodium storage/excretion balance, reflecting salt sensitivity and sodium intake (56). Cardiac contractility is controlled by a combination of sympathetic nerve activity and cardiac function (56). Peripheral vascular resistance is regulated by vascular tone and influenced by vascular remodeling and vasoactive substances such as the renin-angiotensin system (56).An elevated TyG index typically indicates decreased insulin sensitivity, leading to increased IR and blood insulin levels. Impaired insulin sensitivity may heighten the risk of HTN through several mechanisms.

Experimental data suggest that IR and compensated hyperinsulinemia can increase BP through several pathways. First, when insulin resistance leads to hyperinsulinemia, it increases the uptake of glucose and sodium by sodium-glucose cotransporter protein 2 (SGLT2) in the proximal tubule, which ultimately leads to HTN by increasing the volume of circulating fluid (18, 19). Second, the stimulation of sympathetic nerve activity and increased renin excretion (20). This decrease in activity leads to diminished activation of endothelial nitric oxide synthase by insulin, thereby reducing nitric oxide (NO)-mediated vasodilation (21), and ultimately elevating HTN by increasing circulating fluid volume and peripheral vascular resistance. Finally, both hyperglycemia and hyperinsulinemia also promote vascular remodeling and arterial stiffness (22). Insulin resistance results in abnormal mitochondrial biosynthesis and dynamics in endothelial cells, which in turn affects endothelial function, leading to decreased NO production and increased destruction, resulting in an imbalance between endothelium-derived diastolic and systolic factors, increased vascular tone, increased vascular resistance in small peripheral arteries, and ultimately elevated blood pressure (23, 24).

TG is part of the TyG index formula and remains a cardiovascular risk factor, and high plasma concentrations of triglycerides may alter endothelial function and promote cardiac steatosis, myocardial damage, and irreversible cardiac injury (57).TG can be derived from both endogenous and exogenous sources.TG is an ester formed by combining glycerol and three fatty acids (FA), and glucose is the main compound converted to TG (58). Dietary sources are the most important sources of TG. However, TG cannot be absorbed directly through intestinal cells, it is firstly converted to fatty acids and glycerol by specialized lipase and then absorbed through intestinal cells. The transient dynamic process leading to an increase in TG during daily food intake is mainly mediated by insulin, as insulin mediates the synthesis of Apo 48 and the secretion of hepatic very low-density lipoprotein 1 (59).

TG impairs vascular endothelial function through multiple pathways. Firstly, TG is able to act on endothelial cells, inducing vascular smooth muscle cell proliferation/migration and matrix degradation (60). In addition, promotion of inflammatory factors and adhesion molecule expression and reactive oxygen species, as well as recruitment of monocyte-macrophages, enhanced macrophage phagocytosis, and formation of foam cells, which promotes atherosclerosis (61–63).The effect of increased TG-rich lipoprotein (TGRL) lipolysis is another mechanism including altered distribution of zonula occludens-1 and F-actin after TGRL lipolysis (64) and an increase in Caspase-3 activity which triggers nuclear fragmentation and endothelial cell apoptosis (64).The effects of TG and its metabolites on cardiomyocyte function and morphology. Firstly, by altering cardiomyocyte gene expression, which may lead to irreversible cell damage and apoptosis (65). Furthermore, by contributing to an imbalance in ATP synthesis, leading to impaired energy supply and ultimately to a decline in cardiomyocyte contractile dysfunction (66). Although TG maintains the energy supply of cardiac cells through β-oxidation process, high plasma levels of TG as well as TG in muscle cells produce lipotoxic effects (67). In addition, hypertriglyceridemia causes an increase in ceramide concentration in cardiomyocytes by inhibiting Akt signaling and inducing aberrant AMPK biochemical pathways, which can lead to heart failure (68). Kruppel-like factor (KLF) have been suggested as a possible pathophysiology mechanism: dysfunctional KLF 15 promotes impaired lipid utilization, hyperactivity of pro-hypertrophic proteins and stress pathological hypertrophy (69), whereas KLF 4 affects the transcriptional control of cardiac mitochondrial homeostasis (70). In addition, it has been shown that transgenic mice expressing aberrant lipoprotein lipase on the surface of cardiomyocytes accumulate lipids in cardiac muscle cells, which is accompanied by altered morphology and increased size of cardiomyocytes that are unable to provide effective contractile function, thereby inducing ventricular dysfunction (71).

Our study explored two aspects: First, we examined the relationship between the TyG index in childhood and pre-HTN in adulthood, finding that the elevation of the TyG index in childhood was closely related to pre-HTN in adulthood. Changes in the trajectory of the TyG index in childhood had potential diagnostic value for diagnosing BP elevation in adulthood. Second, we conducted a stratified study to determine the role of racial and gender differences in the impact of the childhood TyG index on pre-HTN in adulthood. Based on this study, the scope could be further expanded to include different countries, age groups, or other baseline characteristics. However, the study has some limitations: This is a retrospective study, missing data and confounding factors may introduce bias. Additionally, the sample size for pre-HTN in this study was small, potentially limiting the desired results.

## 5 Conclusions

Our findings suggest that an elevated TyG index in childhood can increase the risk of pre-HTN in adulthood, with significant differences observed by race and gender. Monitoring the dynamics of the TyG index can identify individuals at higher risk of developing pre-HTN, provide early identification of future HTN and other cardiovascular diseases, and reduce the overall incidence of cardiovascular disease by effectively slowing down or stopping the transformation of pre-HTN into HTN by making early preventive plans targeting the TyG index.

## Data Availability

The datasets presented in this study can be found in online repositories. The names of the repository and accession number can be found here: Bogalusa Heart Study (https://biolincc.nhlbi.nih.gov/studies/bhs/).
